# Leveraging large language models to predict antibiotic resistance in *Mycobacterium tuberculosis*

**DOI:** 10.1093/bioinformatics/btaf232

**Published:** 2025-07-15

**Authors:** Conrad Testagrose, Sakshi Pandey, Mohammadali Serajian, Simone Marini, Mattia Prosperi, Christina Boucher

**Affiliations:** Department of Computer and Information Science and Engineering, University of Florida, Gainesville, FL 32611, United States; Department of Computer and Information Science and Engineering, University of Florida, Gainesville, FL 32611, United States; Department of Computer and Information Science and Engineering, University of Florida, Gainesville, FL 32611, United States; Department of Epidemiology, University of Florida, Gainesville, FL 32601, United States; Department of Epidemiology, University of Florida, Gainesville, FL 32601, United States; Department of Computer and Information Science and Engineering, University of Florida, Gainesville, FL 32611, United States

## Abstract

**Motivation:**

Antibiotic resistance in *Mycobacterium tuberculosis* (MTB) poses a significant challenge to global public health. Rapid and accurate prediction of antibiotic resistance can inform treatment strategies and mitigate the spread of resistant strains. In this study, we present a novel approach leveraging large language models (LLMs) to predict antibiotic resistance in MTB (LLMTB). Our model is trained and evaluated on genomic data from 12 185 CRyPTIC isolates and their associated resistance profiles, utilizing natural language processing techniques to capture patterns and mutations linked to resistance. The model’s architecture integrates state-of-the-art transformer-based LLMs, enabling the analysis of complex genomic sequences and the extraction of critical features relevant to antibiotic resistance.

**Results:**

We evaluate our model’s performance using a comprehensive dataset of MTB strains, demonstrating its ability to achieve high performance in predicting resistance to various antibiotics. Unlike traditional machine learning methods, fine-tuning or few-shot learning opens avenues for LLMs to adapt to new or emerging drugs, thereby reducing reliance on extensive data curation. Beyond predictive accuracy, LLMTB uncovers deeper biological insights, identifying critical genes, intergenic regions, and novel resistance mechanisms. This method marks a transformative shift in resistance prediction and offers significant potential for enhancing diagnostic capabilities and guiding personalized treatment plans, ultimately contributing to the global effort to combat tuberculosis and antibiotic resistance.

**Availability and implementation:**

All source code is publicly available at https://github.com/ctestagrose/LLMTB.

## 1 Introduction

*Mycobacterium tuberculosis* (MTB), the causative agent of tuberculosis (TB), is a major global health concern. Reclaiming its position from SARS-CoV-2 (COVID-19), TB is the leading cause of death from a single infectious agent, ranking above HIV/AIDS (World Health Organization 2024). TB caused an estimated 1.25 million deaths in 2023, including 1.09 million among HIV-negative people and 161 000 among people with HIV (World Health Organization 2024). Globally, 175 923 people were diagnosed and treated for multidrug-resistant or rifampicin-resistant TB (MDR/RR-TB), this was 44% of the 400 000 people estimated to have developed MDR/RR-TB in 2023 (World Health Organization 2024), making drug-resistant MTB an urgent growing threat. Drug-resistant TB is driven by a combination of biological and sociological factors, often resulting from the improper use or management of antimicrobial treatments for MTB. This includes, but is not limited to, incorrect drug combinations or inappropriate duration of treatment. The degree of drug resistance in TB can be classified into two major categories: (i) Multidrug-resistant tuberculosis (MDR-TB), defined by resistance to the first-line antibiotics isoniazid (INH) and rifampicin (RIF) (World Health Organization, 2024). (ii) Extensively drug-resistant TB (XDR-TB), characterized by resistance to both INH and RIF along with resistance to a fluoroquinolone and either a second-line or last-resort antibiotic.

Reports of both MDR-TB and XDR-TB have been reported worldwide, posing severe challenges to public health systems worldwide. MTB acquires resistance through spontaneous mutations in its genome, particularly in genes encoding drug targets or drug-activating enzymes. These mutations can be selected and propagated under antibiotic pressure, resulting in the survival and proliferation of resistant strains. The slow growth rate of MTB and its ability to persist in a latent state further complicate the diagnosis and treatment of resistant tuberculosis. Therefore, the timely and accurate identification of resistant MTB strains is crucial. Antibiotic Susceptibility Testing (AST), using agar or broth culturing methods, remains the standard approach to determining antibiotic resistance in MTB isolates (Schön *et al.* 2017, 2020; Procop 2016). Both methods have a propensity to be both time-consuming and resource-intensive (Procop 2016; Schön *et al.* 2017). Molecular techniques, while faster, often require specific knowledge of resistance-conferring mutations, limiting their ability to detect novel or less common genetic mutations. Consequently, there is an urgent need for efficient and accurate automated approaches to predict antibiotic resistance in MTB. Advances in the field of computational biology have shown considerable promise in addressing the urgent need for accurate and rapid AMR predictions in MTB. For instance, TBProfiler (Phelan *et al.* 2019) is one such tool that provides AMR predictions by trimming raw MTB reads with Trimmomatic, aligning them to the H37Rv reference [using BWA (Li and Durbin 2009) or minimap2 (Li 2018)], and calling variants with either Freebayes (Garrison and Marth 2012), GATK (McKenna *et al.* 2010), bcftools (Li 2011), LoFreq (Wilm *et al.* 2012), or Pilon (Walker *et al.* 2014). These variants are then annotated using SnpEff (Cingolani *et al.* 2012) and filtered based on depth, allele frequency, and strand bias, before the pipeline compares these filtered variants to a curated database of resistance variants (including those documented by the World Health Organization) to infer drug resistance profiles.

Alongside TBProfiler, there exist other established tools for MTB AMR prediction. Mykrobe (Hunt *et al.* 2019), for example, constructs a de Bruijn graph from sequencing reads and matches it to a reference graph of known variant probes, using a Poisson-based genotype model to classify variants as resistant or susceptible while filtering out low-confidence calls. Another deterministic tool, ResFinder (Bortolaia *et al.* 2020), uses KMA (Clausen *et al.* 2018) for efficient alignment of raw sequencing reads, avoiding resource-intensive genome assembly. The tool predicts AMR phenotypes based on manually curated gene variants and integrates PointFinder (Zankari *et al.* 2017) for mutation detection. Meanwhile, KvarQ (Steiner *et al.* 2014) adopts a targeted approach by scanning sequencing reads for known drug-resistance mutations and lineage-associated SNPs. Allowing for rapid and high-confidence predictions for key resistance markers like katG and gyrA. Although these tools yield highly accurate results for the antibiotics included in their test suites, a shared challenge among them is the reliance on manually curated databases that may lag behind in capturing novel or less-studied resistance mechanisms, limiting their ability to adapt when new resistance-conferring variants emerge.

Machine learning approaches can alleviate some of these shortcomings by offering the flexibility to learn underlying patterns of resistance without an exclusive dependence on curated lists of known mutations. For example, MTB++ (Serajian *et al.* 2024) uses both Logistic Regression and Random Forest algorithms to predict antibiotic resistance to 13 antibiotics in MTB using the dataset from The CRyPTIC Consortium (2022). While effective at providing highly accurate predictions without the need for an external database, the *k*-mer analysis in MTB++ and other similar models requires extensive computational resources, often involving the ranking and analyzing millions of *k*-mers by frequency and feature importance.

Large Language Models (LLMs) based on transformer architectures (Vaswani *et al.* 2017), such as Bidirectional Encoder Representations from Transformers (BERT) (Devlin *et al.* 2019) offer a promising next step for AMR characterization by enhancing feature extraction and capturing complex genomic signals of resistance, thus reducing the dependence on manual feature engineering. Building on this foundation, we introduce LLMTB, a BERT-based LLM designed to predict AMR of MTB to 13 antibiotics. To our knowledge, LLMTB is the first LLM applied to MTB. The application of LLMs to omics data is not unprecedented; for instance, GenSLM, a hierarchical transformer model leveraging Generative Pre-trained Transformers (GPT) (on individual gene sequences) and stable diffusion, has been a pioneering effort in utilizing LLMs for modeling viral evolution. This illustrates the flexibility of LLMs for specialized genomics contexts.

Our approach uses 6250 MTB isolates for development and compares performance against established predictive tools on a separate test set of 5954 MTB isolates. We evaluate the performance of LLMTB on the binary classification of antibiotic resistance of MTB using precision, recall, F1-score, and area under the receiver operator characteristic (AUCROC) metrics, demonstrating that LLMTB achieves state-of-the-art results, sometimes surpassing existing methods. Additionally, LLMTB employs gene-level tokenization to enhance interpretability and reduce dimensionality, aligning predictions with known biological features. By integrating attention mechanisms, LLMTB highlights relevant genes and intergenic regions, revealing potential novel resistance loci. This interpretable and scalable solution not only improves the efficiency and accuracy of AMR prediction but also uncovers subtle genomic patterns, underscoring the broader potential of LLMs in genomic studies.

## 2 Materials and methods

### 2.1 Data acquisition and genome assembly

The CRyPTIC Consortium (2022) is a global initiative dedicated to understanding and combating drug-resistant tuberculosis. This dataset consists of over 12 000 MTB isolates, each subjected to whole-genome sequencing and tested for minimum inhibitory concentrations against 13 antibiotic drugs (The CRyPTIC Consortium 2022). The drugs included in the CRyPTIC dataset are rifampicin (RIF), isoniazid (INH), ethambutol (EMB), amikacin (AMI), kanamycin (KAN), rifabutin (RFB), levofloxacin (LEV), moxifloxacin (MXF), ethionamide (ETH), linezolid (LZD), clofazimine (CFZ), delamanid (DLM), and bedaquiline (BDQ). In the treatment of TB, first-line antibiotic drugs consist of EMB, INH, and RIF; second-line drugs are AMI, ETH, KAN (injectable agent), LEV, MXF, and RFB; and the last-line are BDQ, CFZ, DLM, and LZD. We downloaded the paired-end high-throughput sequencing data (FASTQ format) for 12 185 MTB isolates provided by the CRyPTIC consortium. These data were accompanied by comprehensive antimicrobial susceptibility testing (AST) tables, which we used to derive precise resistance and susceptibility profiles for each isolate across 13 antibiotics, that is the ground truth for our approach. We assembled all 12 185 whole-genome paired-end datasets using SPAdes (Bankevich *et al.* 2012).

### 2.2 Annotation of genome assemblies

We identified (annotated) all functional sequences and genes from the 12 185 assemblies using Prokka (Seemann 2014), which integrates ab initio gene prediction, sequence similarity searches, and curated databases to deliver comprehensive and accurate annotations. To improve the accuracy of gene annotation, we created a custom protein database based on our set of 16 MTB reference genomes. The annotations for this database were generated using the NCBI Prokaryotic Genome Annotation Pipeline (PGAP) (Tatusova *et al.* 2016). By integrating this custom database into Prokka, we ensured that Prokka prioritized these curated proteins for functional assignments, enabling more specific and comprehensive annotations tailored to the diversity of MTB lineages.

To address fragmented annotations and improve downstream analyses, we developed a post-processing method to merge partial gene alignments into full-length sequences. First, we standardized gene names by aligning Prokka annotations to the H37Rv reference genome and updating FASTA headers for accurate identification. Using BLAT (Kent 2002), query fragments were aligned back to H37Rv gene sequences, and alignment information was processed alongside query sequences. Our algorithm parsed alignment metrics, including alignment length and percent identity, and mapped query identifiers to their sequences for efficient merging. Alignments were clustered by target genes, prioritizing full-length sequences with at least 95% coverage and high percent identity. When full-length alignments were unavailable, fragmented alignments with over 90% identity were retained, sorted, and merged sequentially. Overlapping regions were trimmed, gaps were filled with undefined nucleotides (‘N’s), and sequences were oriented to the positive strand for uniformity, enhancing the dataset’s compatibility with LLMs. The output included a FASTA file of assembled gene sequences and a detailed log of the merging process. To mitigate the impact of these fragmented annotations on downstream analyses, we developed a targeted post-processing method that merges partial gene alignments into full-length sequences.

From this annotated list of genes, we narrowed our focus to 273 genes associated with AMR in MTB, based on data from The CRyPTIC Consortium and The 100 000 Genomes Project (Allix-Béguec et al. 2018), MTB++ (Serajian *et al.* 2024), and the WHO catalog (World Health Organization 2021). This selection aimed to reduce computational overhead while ensuring that the training data were of high quality and highly relevant to AMR.

### 2.3 Identification of intergenic regions

To enhance the model’s ability to recognize gene regulatory elements, we included intergenic regions flanking each of the genes of interest, capturing up to 350-bp upstream and downstream, with a minimum length of 50 bp. This approach accounts for mutations in regulatory elements, such as promoters, that influence AMR, exemplified by the ahpC promoter. Mutations in this region upregulate ahpC expression, compensating for katG loss and contributing to isoniazid resistance (Rinder *et al.* 1998). By including these regions, the model gains insight into subtle mechanisms of resistance. Additionally, genes and their intergenic regions were arranged sequentially to provide spatial context, enabling the model to identify functional relationships or co-regulatory mechanisms critical to AMR.

### 2.4 LLMTB model architecture

**Figure 1.**
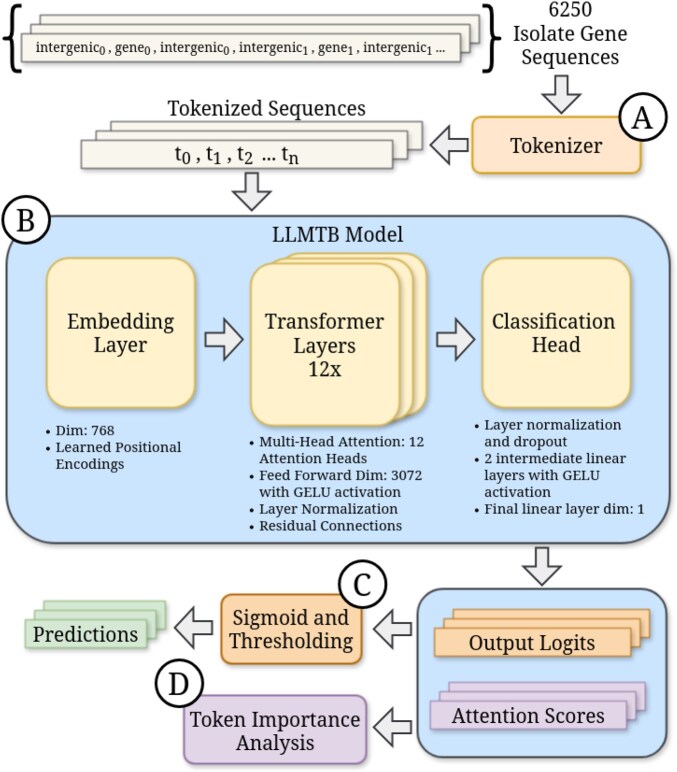
(A) Isolate gene sequences are tokenized where each gene/intergenic region results in a single token. (B) Tokenized sequences are then input to the BERT classification model where resulting logits and attention scores are output. (C) Output logits are fed to a sigmoid function and thresholded to obtain a resulting resistance prediction. (D) Attention scores are used to analyze the important gene or intergenic tokens.

Using PyTorch (Ansel *et al.* 2024), we developed a classification model architecture modeled after BERT (Devlin *et al.* 2019) (Fig. 1 displays a high-level overview). First, tokenized sequences are embedded into 768D vectors, to which learned positional embeddings that preserve the ordering of the tokens are added. The resulting vectors are passed through 12 transformer layers, each consisting of a multi-head attention mechanism with 12 attention heads. A residual connection and layer normalization are applied after the attention mechanism, followed by a feed-forward neural network of dimension 3072. After the feed-forward layer, another residual connection and layer normalization are applied. Following this, the final sequence representations are passed through a pooling layer that pools the outputs from the transformer blocks. These outputs are then fed to a classification head with two intermediate linear layers, dropout, and GELU activations, ultimately outputting a single logit. Output logits are then fed to a sigmoid function to obtain a probability that is then thresholded for a resulting prediction. Alongside the output prediction, the model also outputs attention scores, which are later used to analyze tokens of high importance to the model.

### 2.5 Dataset preparation and LLM training

Of the 12 185 CRyPTIC MTB assemblies, we split them into a training set of 6250 isolates and a test set of 5954, ensuring LLMTB was trained on the same isolates as MTB++ (Serajian *et al.* 2024). After filtering for valid AMR phenotypes, 6224 isolates remained in the training set. To train the LLM, we read annotated gene and intergenic region sequences from each isolate’s FASTA file, filtered them, and sorted them alphanumerically by gene name.

Each gene or intergenic region was then tokenized as a single token, allowing hundreds of sequences to fit within a feasible input length and capturing any haplotype changes as unique tokens. The tokenized data maintained alphanumeric order so that tokens for each gene were aligned across isolates. The set of tokenized isolates was then split into separate training and validation sets on a 4–1 ratio across five folds of the data. For each of the five folds, the binary classification models were trained for 200 epochs using a batch size of 256 on a system with access to 48 CPUs, 128GB of system memory, and 3 NVIDIA A100s. Cosine annealing (Loshchilov and Hutter 2017) was applied in a repeating cycle, where the learning rate was gradually reduced from 2e-4 to 2e-8 over 25 epochs and then increased back to 2e-4 over the next 25 epochs. This pattern was repeated until training was completed, allowing the procedure to leverage the exploration of a larger learning rate and the more refining nature of a smaller learning rate. In order to better train the model in the presence of larger class imbalances, Focal Loss with a dynamic alpha value based on the label frequency was used (Ross and Dollár 2017). Each epoch possessed a validation loop, allowing for the model to be evaluated on its respective validation set and was saved based on the best positive class F1-score on the respective validation set. A single-step adversarial adjustment on the embeddings during training was also used to prevent over-fitting (Miyato *et al.* 2016), alongside dropout and weight decay. Following the training procedure, models for each antibiotic were trained using parameters determined from the five-fold cross-validation procedure. These models were then applied to the hold-out test set and compared to MTB++ (Serajian *et al.* 2024), TBProfiler (Phelan *et al.* 2019), Resfinder (Bortolaia *et al.* 2020), Mykrobe (Hunt *et al.* 2019), and KVarQ (Steiner *et al.* 2014).

## 3 Results

### 3.1 Results incorporating intergenic regions

**Figure 2.**
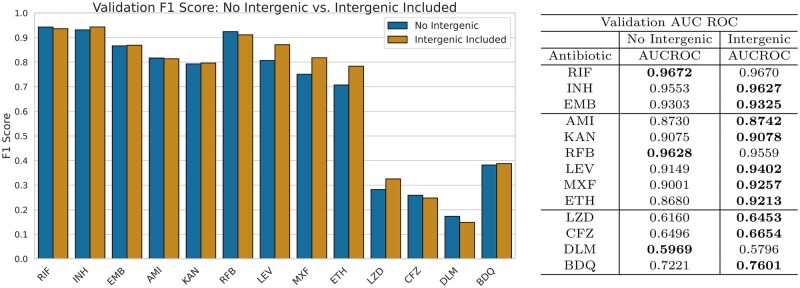
Comparison of average validation F1-scores and AUCROC scores for BERT classification models with and without intergenic regions across five folds. The results illustrate the impact of including intergenic regions on the predictive performance for various antibiotics.

LLMTB’s performance on a validation set was used to identify and refine the best-performing models. Also examined was the effect of the inclusion of variable-length intergenic regions alongside their corresponding genes on model performance. Figure 2 shows the average F1-scores across 13 antibiotics for LLMTB over five folds of training/validation data, comparing results with and without intergenic regions.

For first-line antibiotics, LLMTB achieves the highest average F1-scores of 0.9428, 0.9432, and 0.8689 on RIF, INH, and EMB, respectively. Notably, all first-line antibiotics yield AUCROC scores above 0.93, demonstrating the model’s strong ability to distinguish between resistant and non-resistant isolates. While excluding intergenic regions slightly improved performance on RIF (0.9359–0.9428), including them led to higher F1-scores on INH (0.9312–0.9432) and had a small positive impact on EMB (0.8661–0.8689).

On second-line antibiotics, LLMTB attains top average F1-scores of 0.8139, 0.7964, 0.9240, 0.8708, 0.8179, and 0.7834 on AMI, KAN, RFB, LEV, MXF, and ETH, respectively. Except for AMI (AUCROC 0.8742), all second-line antibiotics have AUCROC values above 0.90. Among these, the inclusion of intergenic regions substantially boosts the average F1-score and AUCROC for LEV (0.8065–0.8708), MXF (0.7508–0.8179), and ETH (0.7073–0.7834). The inclusion of intergenic regions provided a minor improvement to the F1-score for KAN (0.7929–0.7964), whereas excluding intergenic regions benefited RFB (0.9112–0.9240). It is worth noting that the data imbalance present in second-line antibiotics poses an additional challenge when predicting resistance to these drugs.

When compared to first- and second-line antibiotics, the average validation performance of LLMTB on last-line antibiotics is substantially reduced. LLMTB provides the best F1-scores of 0.3253, 0.2591, 0.1732, and 0.3875 on LZD, CFZ, DLM, and BDQ, respectively. The AUC ROC scores for all last-line antibiotics are greater than 0.59, with BDQ achieving the highest AUC ROC of 0.76. The inclusion of intergenic regions provided an increase in F1-score for LZD (0.2822–0.3253) and BDQ (0.3817–0.3875) while their exclusion increased the F1-score for CFZ (0.2478–0.2591) and DLM (0.1487–0.1732).

### 3.2 Evaluation of LLMTB against competing methods

LLMTB’s performance on the 5954 hold-out test isolates was compared with that of MTB++ (Serajian *et al.* 2024), Resfinder (Bortolaia *et al.* 2020), TBProfiler (Phelan *et al.* 2019), Mykrobe (Hunt *et al.* 2019), and KvarQ (Steiner *et al.* 2014). Separate LLMTB models were trained for each antibiotic and applied to the test set; the same test set was also evaluated with all competing tools. Table 1 outlines the F1-score of each of these tools on the hold-out set of isolates.

**Table 1.** Positive class F1-scores for LLMTB, MTB++, Resfinder, TBProfiler, Mykrobe, and KvarQ on the hold out test set of 5,954 isolates. The highest F1-score for each antibiotic is shown in bold.

Method	RIF	INH	EMB	AMI	KAN	RFB	LEV	MXF	ETH	LZD	CFZ	DLM	BDQ
LLMTB	0.9094	0.9361	0.8273	0.8153	0.7005	**0.8913**	0.8440	0.7856	0.7562	0.4466	**0.3074**	0.0862	0.2095
MTB++	0.8986	0.9490	0.7889	0.7952	0.7025	0.8879	0.8505	0.7337	0.6845	0.1481	0.0380	0.0000	0.0000
Resfinder	0.9323	0.9535	0.8428	0.8176	0.6998	N/A	N/A	N/A	0.7181	0.4536	0.0070	N/A	0.0769
TBProfiler	**0.9362**	0.9624	**0.8512**	0.7958	0.7041	N/A	**0.8688**	**0.7940**	**0.7818**	**0.5098**	0.2217	**0.2581**	**0.2594**
Mykrobe	0.9339	**0.9625**	0.8489	0.7905	**0.7138**	N/A	0.8635	0.7925	0.7122	0.4211	N/A	0.1687	N/A
KVarQ	0.9180	0.9415	0.7812	**0.8199**	0.6953	N/A	N/A	N/A	N/A	N/A	N/A	N/A	N/A
Δ	0.0296	0.0264	0.0264	0.0046	0.0133	0.0000	0.0248	0.0084	0.0256	0.0898	0.0000	0.1923	0.0499

Delta between LLMTB and the best performing method is shown in bottom-most row.

MTB++ is trained on the same 6250 isolates as LLMTB and utilizes both logistic regression and random forest models to provide its predictions. Comparing the test set performance of LLMTB to that of MTB++, LLMTB achieves higher F1-scores on 10 of the 13 antibiotic drugs. Among the first-line antibiotics, LLMTB outperforms MTB++ on RIF and EMB, while MTB++ has a slight advantage on INH. For the second-line antibiotics, LLMTB surpasses MTB++ on AMI, RFB, MXF, and ETH. However, MTB++ performs better on KAN and LEV when compared to LLMTB. Notably, for last-resort antibiotics, LLMTB dramatically outperforms MTB++. On both DLM and BDQ, LLMTB outperformed MTB++, which provided F1 scores of 0.00. LLMTB does achieve better F1-scores on the last-resort antibiotics LZD and CFZ. It should be emphasized that LLMTB and MTB++ were both trained on the same set of 6224 CRyPTIC isolates, therefore highlighting LLMTB’s ability to generalize effectively on new data.

Alongside the machine-learning-based MTB++, LLMTB was also compared to the deterministic tools Resfinder, TBProfiler, Mykrobe, and KvarQ. Of the tools compared, only LLMTB and MTB++ provide predictions for RFB, with LLMTB yielding a higher F1-score. TBProfiler predicts resistance for 12 of the 13 antibiotics, Mykrobe predicts for 10, and KvarQ predicts for 5. On both first- and second-line antibiotics, LLMTB remains within 3 points of the best-performing method’s F1-score. TBProfiler achieves the best F1-score on RIF, EMB, LEV, MXF, ETH, LZD, DLM, and BDQ. MyKrobe achieves the best F1-score on both INH and KAN. KvarQ achieves the best F1-score on AMI.

On the last-line antibiotics (LZD, CFZ, DLM, and BDQ), all tools that offered predictions exhibited reduced classification performance relative to first- and second-line antibiotics. LLMTB achieves the highest F1-score on CFZ, followed by TBProfiler. Except for KvarQ, all tools make predictions for LZD; TBProfiler performs best, while LLMTB exceeds the results of both MTB++ and Mykrobe. For DLM, predictions are available from all but Resfinder and KvarQ; TBProfiler again achieves the top F1-score, outperforming the other methods, including LLMTB. On BDQ, LLMTB attains an F1-score behind only TBProfiler.

TBProfiler’s success on 8 of the 13 antibiotics can be attributed to its static, manually curated database of WHO-cataloged mutations, which includes the CRyPTIC isolates. Similar curated databases used by Mykrobe, KvarQ, and Resfinder help ensure high specificity and strong clinical relevance but also require labor-intensive updates, potentially delaying adaptation to emerging resistance mechanisms.

In contrast, LLMTB leverages a retrainable architecture based on large-scale gene sequence embeddings and a transformer-based BERT model. Since it does not rely on a fixed, manually curated database, LLMTB can easily incorporate newly sequenced isolates, thereby staying current with emerging resistance variants.

Additionally, the deterministic tools compared in this study may overlook novel or slightly divergent genes, whereas LLMTB’s ability to generalize across varied genomic contexts enables it to detect resistance mechanisms not explicitly cataloged. Despite lacking a large predefined database, LLMTB delivers performance comparable to database-driven methods, underscoring the dynamic capacity of large language models to generalize across extensive and previously unseen sequence data.

### 3.3 Resource usage across tools

In addition to comparing the classification performance between LLMTB and the other established tools, the computational resource requirements for prediction were also examined. The resource usage of all methods was monitored on a system with 48 CPU cores, 128 GB of RAM, and an Nvidia A100 GPU (80 GB of VRAM), using the same test set of 5954 isolates (Table 2).

**Table 2.** Resource usage for LLMTB, MTB++, Resfinder, TBProfiler, Mykrobe, and KvarQ.

Maximum resource usage for prediction							
	LLMTB preprocessing	LLMTB Prediction	MTB++	Resfinder	TBProfiler	Mykrobe	KVarQ
Time	09:28:49	00:26:21	02:10:30	02:11:28	13:06:31	08:08:29	14:10:57
Memory	13.04 GB	3.18 GB	5.68 GB	6.24 GB	112.19 GB	26.83 GB	1.79 GB
VRAM	N/A	6.00 GB	N/A	N/A	N/A	N/A	N/A

Each method was run with access to 48 CPUs and 128 GB of RAM.

LLMTB has a preprocessing step where the genes and intergenic regions are extracted from the isolate assemblies, with this step taking approximately 9.5 h and a maximum of 13 GB of RAM. It is worth noting that most of this processing time comes from the annotations of the genes using Prokka. The subsequent predictive step completes in roughly 26 min using a maximum of 3.18 GB of RAM and 6.00 GB of VRAM. Among the other tools tested, KvarQ has the smallest RAM footprint (1.79 GB) yet requires over 14 h to complete. TBProfiler has the highest memory requirement, needing 112 GB of RAM, and takes over 13 h to run to completion. By comparison, Resfinder and MTB++ both possess runtimes under 2.5 h, and both use less than 6.5 GB of RAM. Mykrobe ran in under 9 h but required a maximum of 26.8 GB of RAM, positioning it in the mid-range for overall resource needs. These findings underscore the trade-offs between resource usage and runtime across different approaches. Notably, LLMTB achieves performance comparable to TBProfiler while consuming considerably less RAM and completing its analysis much faster.

### 3.4 Model explainability

LLMs utilize tokenization to split sequences into manageable units and employ attention mechanisms to focus on relevant features, enabling interpretable predictions. LLMTB’s attention mechanism and tokenization strategy allow us to determine genes and intergenic regions that significantly influence our model’s predictions. To quantify the importance of these features, attention scores were extracted from the multi-head attention mechanism in the transformer block from each layer and averaged across the layers to determine a per-token average attention score. We then calculate gene and intergenic region importance by averaging the tokens associated with each gene and intergenic region. Additionally, we examine the top 10 genes and intergenic regions with the maximum attention scores in each fold. To evaluate their importance, we apply three complementary metrics—Max, Average, and *Z*-score, each offering a unique perspective for interpretation. This is critical for model explainability, as it helps uncover novel resistance mechanisms and enhances our understanding of resistance at the nucleotide sequence level.

**Figure 3.**
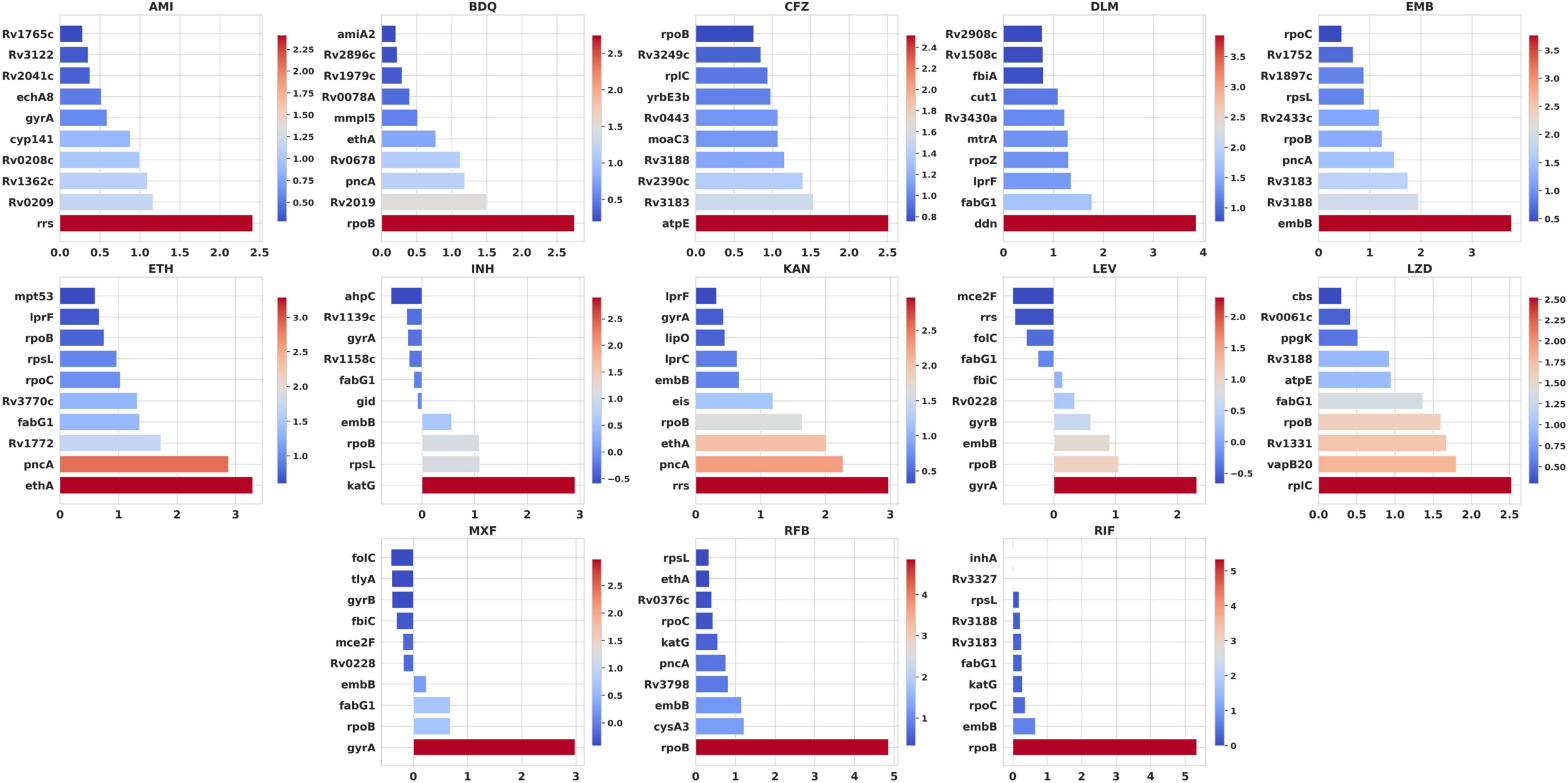
Consolidated bar chart illustrating the top 10 genes ranked *Z*-score of average attention scores across five cross-validation folds. The y-axis represents the genes, while the *x*-axis represents the Z-scores of their attention scores. The color gradient reflects the magnitude of the *Z*-scores, highlighting genes with higher attention scores more prominently.

When examining the top-ranked genes for each drug across the three attention-based metrics, our model (LLMTB) consistently identifies canonical resistance determinants while also highlighting less-characterized genes (Fig. 3; Supplementary Figs S3 and S4). The Max metric captures instances in which a gene has exceptionally high importance in one or more folds; in contrast, the Average metric aggregates a gene’s relevance across all folds, offering a more stable measure of overall importance. Finally, the *Z*-score normalizes each gene’s average importance relative to the global distribution, emphasizing genes that stand out statistically from the mean.

Although these three metrics often converge on the same core resistance genes, certain instances reveal unique advantages and limitations. For example, in BDQ resistance, atpE and Rv0678 [both known to mediate resistance through efflux pump regulation (Nguyen *et al.* 2018)] prominently feature in the Max and Average rankings. However, the *Z*-score metric does not contain atpE at all, likely due to the highly skewed distribution of attention scores in imbalanced datasets. Because the *Z*-score highlights genes with unusually large average contributions relative to the overall variation, it may under-identify consistently moderate-score genes in cases where the model encounters fewer resistant examples. This highlights the importance of utilizing complementary metrics like Max and Average to capture different facets of gene relevance.

Across all three metrics, the top-ranked genes for the remaining antibiotics align closely with well-established resistance targets and pathways (World Health Organization 2023). For the injectable drugs AMI and KAN, resistance primarily relies on mutations in the rrs gene (Alangaden *et al.* 1998), which encodes the 16S rRNA. AMI targets the 16S rRNA (Alangaden *et al.* 1998), disrupting protein synthesis by binding to conserved regions. Similarly, KAN targets both the 16S rRNA and the broader 30S ribosomal subunit (Knox *et al.* 2024), providing an overlapping mechanism of action. Another gene of importance in KAN resistance is *eis* (Gikalo *et al.* 2012), which encodes an aminoglycoside acetyltransferase. Mutations in the promoter region of *eis* lead to its overexpression, enabling the enzyme to acetylate and inactivate KAN (Zaunbrecher *et al.* 2009a). This resistance mechanism can be used to distinguish strains resistant to kanamycin (Zaunbrecher *et al.* 2009b). Interestingly, LLMTB also identified *eis* as a gene of significance, demonstrating its ability to align with established resistance mechanisms while independently highlighting key contributors to antibiotic resistance.

Similarly, for the fluoroquinolones LEV and MXF, LLMTB strongly associated resistance with the DNA Gyrase subunits and their intergenic regions, consistent with their established roles as targets of fluoroquinolone therapy. Mutations in these gyrase-encoding genes disrupt the drugs’ ability to inhibit DNA replication (Hooper 1999). Building on this, the model also captured key determinants for INH resistance. For INH, the model highlighted katG, inhA, rpsL, and rpoB corresponding to the canonical mechanisms of INH activation and target inhibition (World Health Organization 2021). For RIF and RFB, rpoB was dominant, aligning with the extensively characterized rifamycin resistance mutations, in the rpoB “RIF resistance-determining region” (Anthony *et al.* 2005).

This trend of accurately associating key resistance genes with their corresponding drugs highlights the model’s consistency and alignment with established resistance pathways. For example, EMB was predominantly linked to embB, while ETH was primarily associated with ethA and inhA, consistent with established literature and WHO guidelines on primary resistance mutations (World Health Organization 2021).

For last-resort antibiotics, the model also demonstrated strong concordance with known resistance loci. BDQ prominently featured atpE, Rv0678, and mmpS5, all of which are well-documented in BDQ-resistant clinical isolates (World Health Organization 2021). For DLM, the model consistently highlighted ddn, in alignment with its activation pathway (World Health Organization 2021). Similarly, LZD was strongly associated with ribosomal protein genes such as rplC, reflecting established mechanisms of LZD resistance (World Health Organization 2021).

These findings are further supported by the intergenic regions (Supplementary Figs S5–S7), where high attention scores before and after genes of interest indicate that gene regulation plays a role in MTB’s resistance mechanisms. The consistent identification of core resistance genes and intergenic regions across multiple metrics underscores LLMTB’s ability to accurately capture the established biology of drug resistance in MTB.

Interestingly, certain genes emerged prominently despite not being the primary resistance determinants, highlighting the model’s ability to capture overlapping or secondary resistance mechanisms. For instance, pncA consistently ranked highly for ETH, BDQ, and KAN resistance in both the Max and Average metrics, even though it is not involved in their drug pathways. This aligns with the notion that resistance to pyrazinamide, a first-line drug, may be present in isolates already treated with or resistant to second- and last-line antibiotics.

Additionally, the importance of genes not widely implicated in resistance, such as vapB20 under LZD, suggests that the model may be uncovering novel associations. The VapBC20 toxin-antitoxin (TA) system, to which VapB20 belongs, is known to contribute to bacterial persistence rather than direct resistance (Winther *et al.* 2013). VapC20, the toxin counterpart, cleaves the sarcin–ricin loop of 23S rRNA, leading to translational arrest and enabling the formation of persister cells under stress conditions (Winther *et al.* 2013). Such persistence mechanisms may indirectly affect antibiotic efficacy, including that of LZD, by allowing subpopulations of bacteria to survive prolonged exposure. This finding highlights the model’s potential to identify genes involved in secondary or indirect resistance pathways, which are critical for understanding treatment challenges in MTB.

These findings underscore the potential of LLMTB to advance our understanding of both well-established and emerging antimicrobial resistance (AMR) mechanisms in MTB. Unlike existing methods, LLMTB uniquely excels in identifying novel genes, uncovering the significance of mutations in regulatory regions, and detecting previously unrecognized secondary resistance mechanisms. This comprehensive capability sets LLMTB apart, providing a deeper and more nuanced perspective on AMR pathways in MTB.

## 4 Discussion

This study examined the development of an LLM for the task of predicting AMR to 13 different antibiotic drugs in MTB genomic sequencing data. Our method, LLMTB, was developed using 6250 isolates from the CRyPTIC dataset of 12 185 MTB isolates. We extract genes and their corresponding upstream–downstream intergenic regions from each isolate’s DNA sequences. These genes and intergenic regions are then individually tokenized via a method of tokenization where each gene or intergenic region corresponds to a single token. One notable advantage of using gene-level tokenization is the enhanced interpretability and reduced dimensionality compared to traditional *k*-mer methods. Each gene and its intergenic region are represented as a discrete token, which naturally aligns with established biological features associated with AMR. This approach can more readily capture structural changes or large deletions affecting entire genes, rather than relying on overlapping substrings that may not directly map to known loci.

Through five-fold cross-validation, we were able to show that LLMTB, on average, is able to achieve exceptional results on AMR prediction in MTB. Where other machine learning tools that utilize extensive *k*-mer analysis may take weeks to develop on high-performance computing architectures, five-fold cross-validation of LLMTB takes roughly 1 day when including the data preprocessing steps. Alongside the rapid training of the model, LLMTB achieves classification results in line with those of the highly accurate tool TBProfiler on a test set of 5954 unseen isolates. This demonstrates that an LLM trained on extracted genes can achieve results comparable to other established deterministic tools. These tools use databases of mutations and lineages associated with antibiotic resistance, increasing the amount of effort required to maintain the clinical relevance of these deterministic prediction tools, especially on novel or unpublished resistance mechanisms. MTB++, while foregoing the need for a maintained database in favor of traditional machine learning algorithms, the need for extensive *k*-mer frequency and feature analysis increases the required development time and resources due to the filtering and ranking of millions of unique *k*-mers. Furthermore, MTB++ appears to struggle on more than half of the antibiotics (EMB, AMI, MXF, ETH, LZD, CFZ, DLM, and BDQ) when applied to the holdout data set of 5954 isolates, where LLMTB provides more competitive results. Despite these strong results, we observed reduced performance for certain last-line antibiotics, primarily due to significant class imbalance and possible unknown resistance mechanisms. In our future work, we aim to address these limitations and more effectively capture mechanisms of AMR, which may not be solely gene-based, by expanding the model’s contextual understanding to whole-genome data while maintaining the scalability of our model.

However, these constraints limit the attention mechanism and positional encodings in LLMTB, allowing the capture of nuanced interactions between genes, potentially leading to more consistent and improved results on unseen or novel data. In addition, we are able to leverage the attention mechanism and determine the genes and intergenic regions that are of importance to the model, offering a higher level of model explanability and interpretation of the results. For example, preliminary attention analysis revealed high-weight signals in genes previously identified as conferring resistance, which validates LLMTB and highlights opportunities to discover unique or novel resistance mechanisms that should be subjected to more scrutiny.

## 5 Conclusion

A key innovation of LLMTB is the inclusion of intergenic regions, which allows regulatory elements of the analysis that influence resistance and expands the genomic scope of the analysis beyond the coding regions. To address challenges such as data scarcity and prediction uncertainty, future work will integrate a rule-based alignment method and a Retrieval-Augmented Generation (RAG) framework, enabling LLMTB to dynamically learn from new genomic data and adapt to emerging resistance mechanisms. Additionally, expanding the training dataset to include more isolates and antibiotics, along with enhancing the models’ adaptability through advanced architectures, broader gene sets, and comprehensive multi-species AMR datasets, will further improve generalization. Lastly, we also plan to investigate the use of LLMTB for the prediction of MIC values alongside binary resistance profiles.

## Supplementary data

Supplementary data are available at *Bioinformatics* online.

## Funding

This research was funded by NIH/NIAID (Grant No. R01AI14180), NSF/SCH (Grant No. INT-2013998), and by the Artificial Intelligence and Complex Computational Research Award.

## Supplementary Material

btaf232_Supplementary_Data

## Data Availability

All data supporting the findings of this study are publicly available from the sources cited in the manuscript.
