# Supplementary material for: Leveraging large language models to predict antibiotic resistance in Mycobacterium tuberculosis
Source: Bioinformatics. 2025 Jul 15;41(Suppl 1):i40–8. doi: 10.1093/bioinformatics/btaf232 (PMC12261485; doi:10.1093/bioinformatics/btaf232)
Supplement: btaf232_Supplementary_Data [file btaf232_supplementary_data.pdf]

SUPPLEMENT

Reference Name	NCBI ID	Location Isolated
F11	NZ_KK339377.1	South Africa
Erdman	NC_020559.1	United States
H37Rv	NC_000962.3	United States
H37Ra	NC_009525.1	United States
CDC1551	NZ_KK341227.1	United States
K Strain	NZ_CP007803.1	Korea
TCDC07	NZ_CP047163	Taiwan
TCDC10	NZ_CP047164.1	Taiwan
TCDC11	NZ_CP046728.2	Taiwan
TBMENG-03	NZ_CP029065.1	India
Beijing	NZ_CP011510.1	China
C2	NZ_JMEK01000001.1	India
HN506	NZ_AP018036.1	Vietnam
KZN1435	NC_012943.1	South Africa
OSDD071	NZ_AHHX01000660	India
OSDD504	NZ_AHHY01001040	India

Table S1. Reference genomes used to assess the assembly quality.

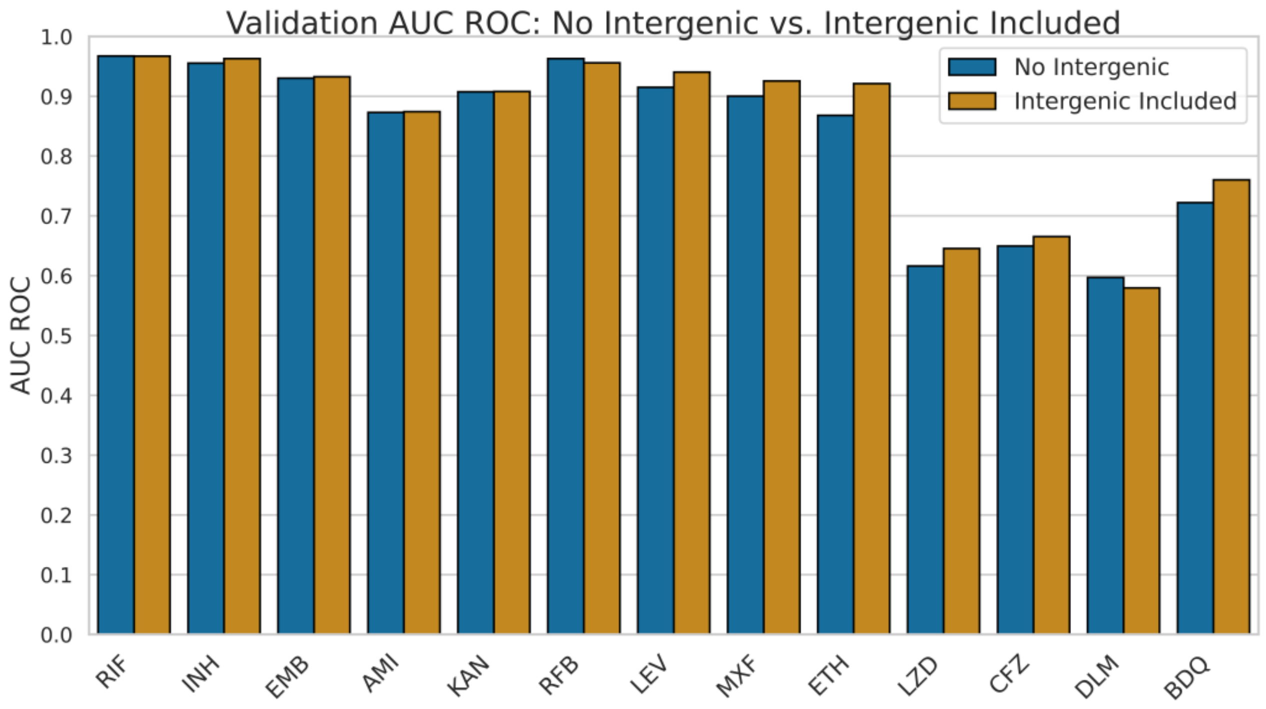

Fig. S1. Average validation F1-scores.

LLMTB Validation F1 No Intergenic vs. Intergenic						
	No Intergenic			Intergenic		
Antibiotic	Precision	Recall	F1	Precision	Recall	F1
RIF	0.9412	0.9444	0.9428	0.9283	0.9436	0.9359
INH	0.9477	0.9153	0.9312	0.9539	0.9327	0.9432
EMB	0.8617	0.8708	0.8661	0.8696	0.8685	0.8689
AMI	0.8916	0.7490	0.8139	0.8916	0.7490	0.8139
KAN	0.8890	0.7165	0.7929	0.8654	0.7408	0.7964
RFB	0.9184	0.9297	0.9240	0.9210	0.9019	0.9112
LEV	0.7955	0.8198	0.8065	0.8630	0.8798	0.8708
MXF	0.7178	0.7889	0.7508	0.7701	0.8727	0.8179
ETH	0.7188	0.6974	0.7073	0.7731	0.7962	0.7834
LZD	0.7129	0.1875	0.2822	0.7341	0.2250	0.3253
CFZ	0.2584	0.2640	0.2591	0.2593	0.2480	0.2478
DLM	0.1840	0.2435	0.1732	0.1960	0.1391	0.1487
BDQ	0.5615	0.3555	0.3817	0.4491	0.3777	0.3875

Table S2. Average validation precision, recall, and F1-score (positive class) for the BERT classification model with and without intergenic regions across 5 folds.

Maximum Resource Usage Training		
	LLMTB Preprocessing	LLMTB 5 Fold Cross Validation
Time	08:00:39	17:33:24
Memory	13.46 GB	46.22 GB
VRAM	N/A	34.44 GB (Per GPU Rank)

Table S3. Resource usage for LLMTB training and preprocessing of training data. Resource usage for LLMTB training represents 5 folds of the dataset for all 13 antibiotics.

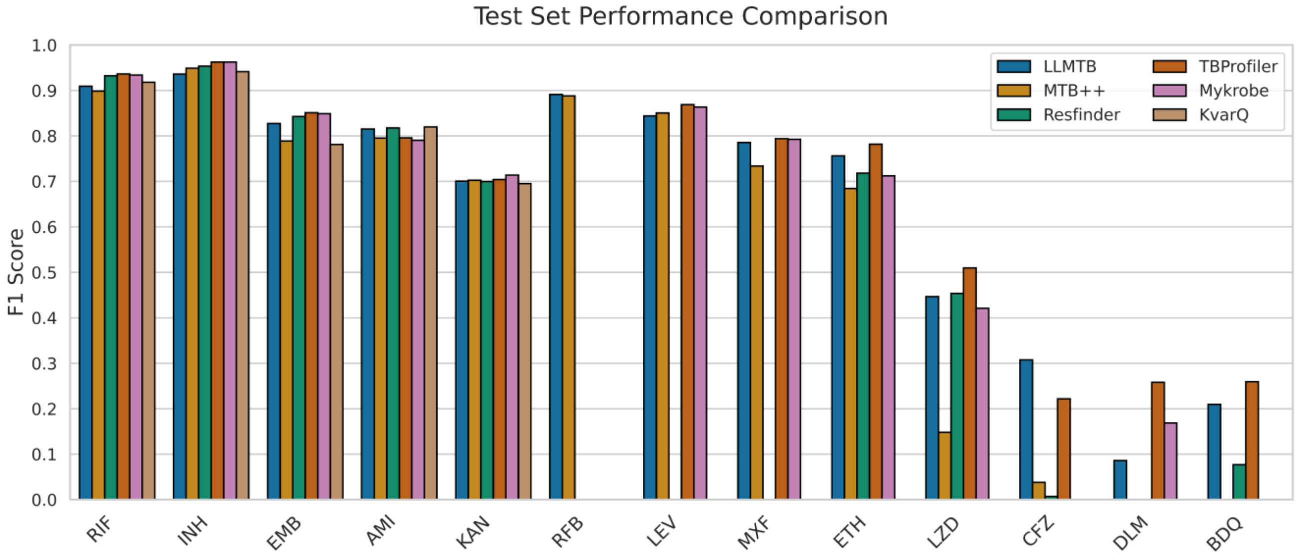

Fig. S2. F1-scores of each of the predictive tools (, MTB++, Resfinder, TBProfiler, Mykrobe, KvarQ) on the test set of 5,954 MTB isolates.

Test Set Precision and Recall												
	LLMTB		MTB++		Resfinder		TBProfiler		Mykrobe		KVarQ	
Antibiotic	Precision	Recall	Precision	Recall	Precision	Recall	Precision	Recall	Precision	Recall	Precision	Recall
RIF	0.8956	0.9237	0.9064	0.8909	0.9071	0.9590	0.8990	0.9766	0.8996	0.9709	0.9042	0.9322
INH	0.9538	0.9190	0.9744	0.9249	0.9700	0.9375	0.9699	0.9550	0.9787	0.9469	0.9840	0.9025
EMB	0.8086	0.8468	0.7250	0.8652	0.8074	0.8815	0.8108	0.8958	0.8275	0.8714	0.8438	0.7272
AMI	0.8333	0.7980	0.8455	0.7506	0.8207	0.8145	0.7445	0.8546	0.7489	0.8371	0.8359	0.8045
KAN	0.7982	0.6241	0.7838	0.6365	0.6538	0.7527	0.6482	0.7705	0.6773	0.7544	0.8448	0.5907
RFB	0.9048	0.8782	0.9075	0.8690	N/A	N/A	N/A	N/A	N/A	N/A	N/A	N/A
LEV	0.8361	0.8520	0.8357	0.8658	N/A	N/A	0.8221	0.9211	0.8367	0.8921	N/A	N/A
MXF	0.7143	0.8727	0.7163	0.7520	N/A	N/A	0.6836	0.9470	0.6958	0.9203	N/A	N/A
ETH	0.7955	0.7207	0.8195	0.5877	0.7974	0.6532	0.7723	0.7915	0.7977	0.6433	N/A	N/A
LZD	0.8214	0.3067	1.0000	0.0800	0.9167	0.3014	0.8966	0.3562	0.9091	0.2740	N/A	N/A
CFZ	0.3264	0.2904	0.0729	0.0257	0.0667	0.0037	0.2865	0.1808	N/A	N/A	N/A	N/A
DLM	0.0658	0.0658	0.0000	0.0000	N/A	N/A	0.6316	0.1622	0.7778	0.0946	N/A	N/A
BDQ	0.2683	0.1719	0.0000	0.0000	0.2000	0.0476	0.1761	0.4921	N/A	N/A	N/A	N/A

Table S4. Precision and recall for LLMTB, MTB++, Resfinder, TBProfiler, Mykrobe, and KvarQ on the hold out test set of 5,954 isolates.

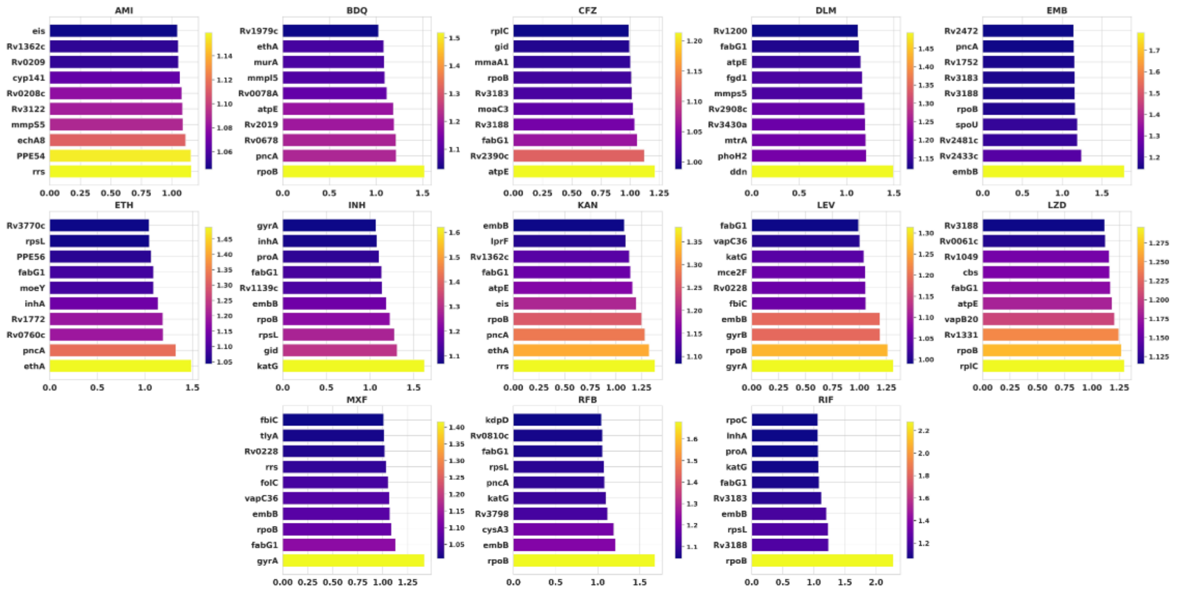

Fig. S3. Consolidated bar chart of the top 10 genes with the highest maximum attention scores observed across five cross-validation folds. By focusing on the peak attention scores, this visualization identifies genes that consistently exhibit strong influence within the model.

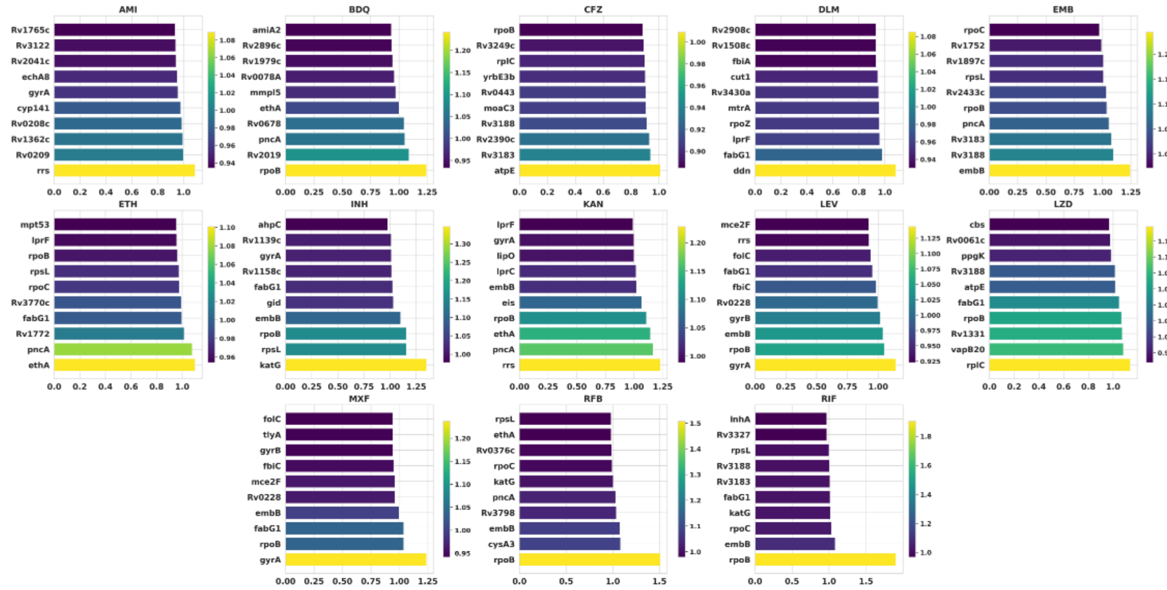

Fig. S4. Consolidated bar chart of the top 10 genes with the highest average attention scores across five cross-validation folds. The average attention score for each gene was computed by taking the mean of its attention scores from all folds.

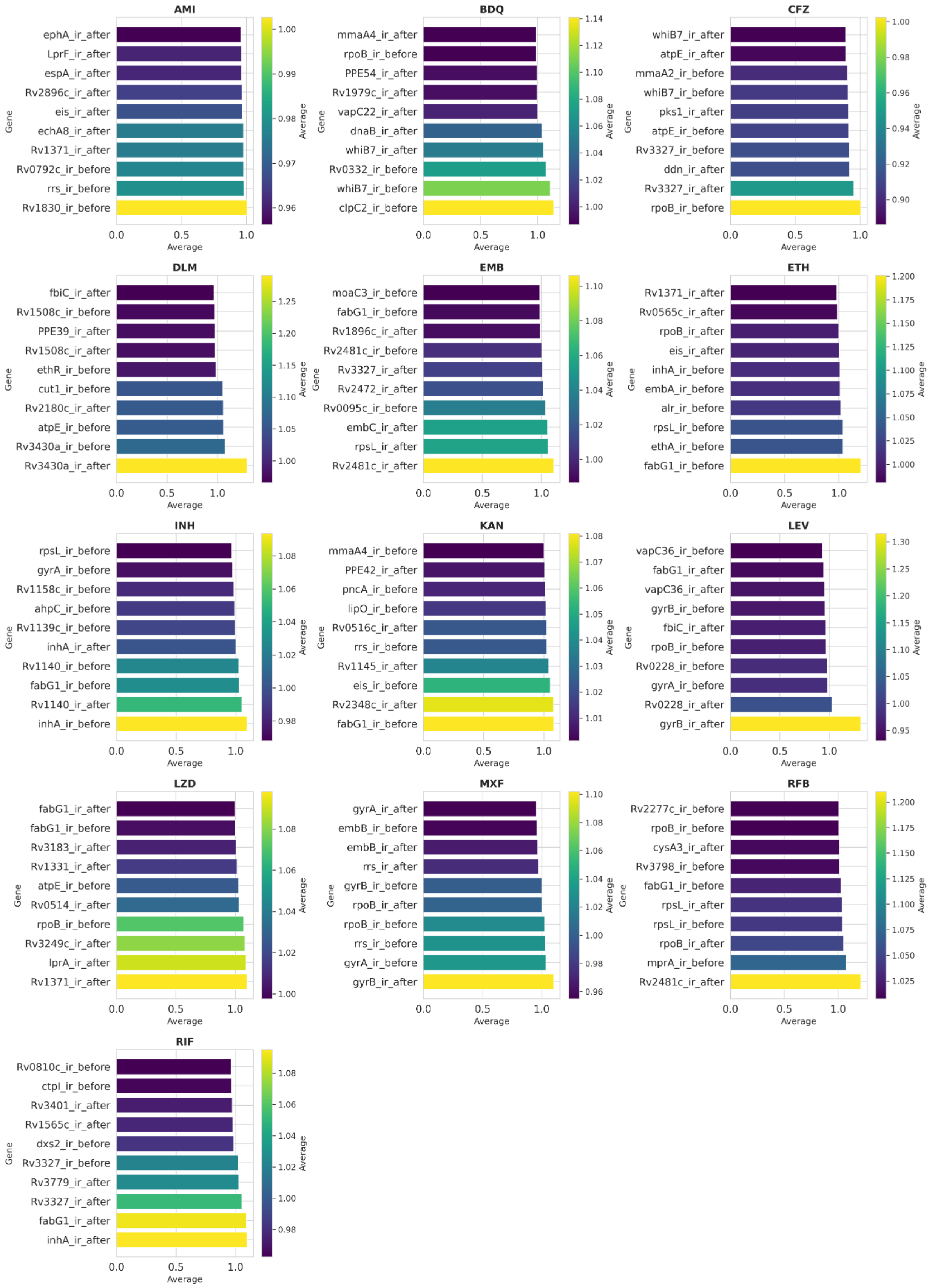

Fig. S5. Consolidated bar chart of the top 10 intergenic regions(IR) with the highest average attention scores across five cross-validation folds. The average attention score for each IR was computed by taking the mean of its attention scores from all folds.

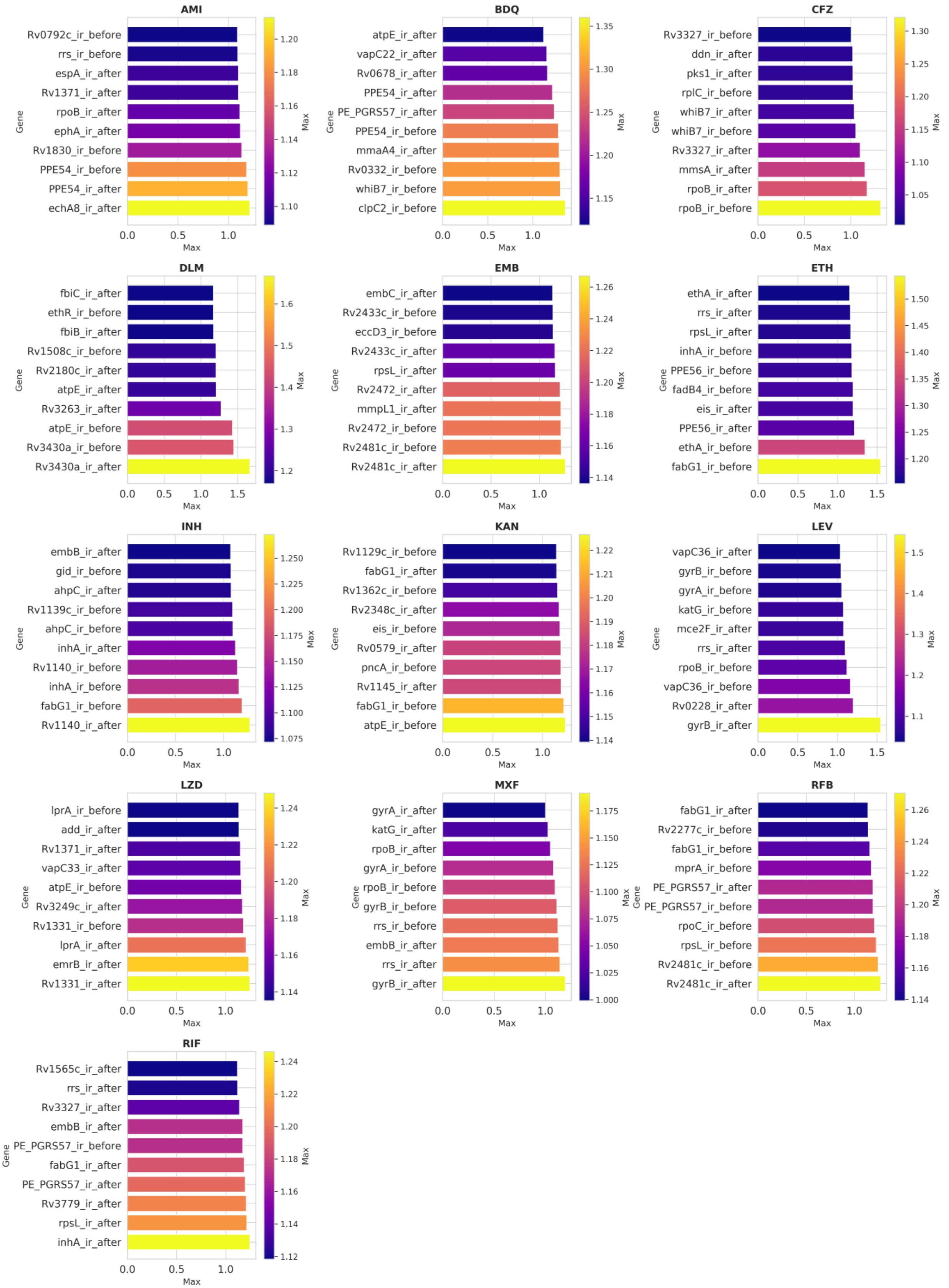

Fig. S6. Consolidated bar chart of the top 10 intergenic regions(IR) with the highest maximum attention scores observed across five cross-validation folds.By focusing on the peak attention scores, this visualization identifies IR that consistently exhibit strong influence within the model.

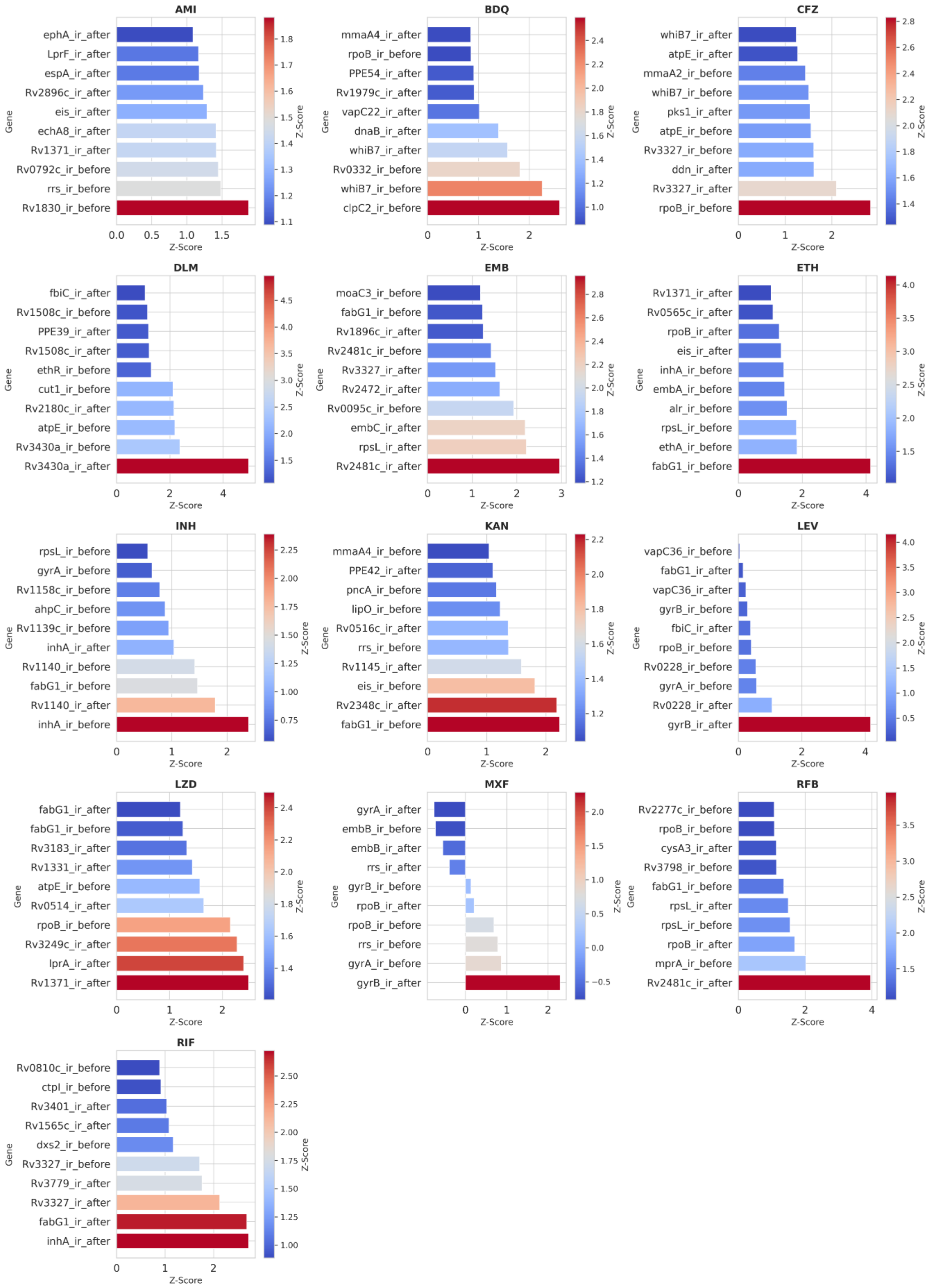

Fig. S7. Consolidated bar chart illustrating the top 10 IR ranked by the Z-score of average attention scores across five cross-validation folds.
